# Astronomical Observations in Support of Planetary Entry-Probes to the Outer Planets

**DOI:** 10.1007/s11214-024-01080-3

**Published:** 2024-06-11

**Authors:** Bonnie J. Buratti, Glenn S. Orton, Michael T. Roman, Thomas Momary, James M. Bauer

**Affiliations:** 1 Jet Propulsion Laboratory California Institute of Technology, Pasadena, CA USA; 2 University of Leicester, Leicester, UK; 3 University of Maryland, College Park Maryland, USA

**Keywords:** Planetary probes, Saturn, Jupiter, Neptune, Uranus

## Abstract

A team of Earth-based astronomical observers supporting a giant planet entry-probe event substantially enhances the scientific return of the mission. An observers’ team provides spatial and temporal context, additional spectral coverage and resolution, viewing geometries that are not available from the probe or the main spacecraft, tracking, supporting data in case of a failure, calibration benchmarks, and additional opportunities for education and outreach. The capabilities of the support program can be extended by utilizing archived data. The existence of a standing group of observers facilitates the path towards acquiring Director’s Discretionary Time at major telescopes, if, for example, the probe’s entry date moves. The benefits of a team convened for a probe release provides enhanced scientific return throughout the mission. Finally, the types of observations and the organization of the teams described in this paper could serve as a model for flight projects in general.

## Introduction

The giant planets of the Solar System have been key targets in past and future missions, starting with the Pioneer and Voyager spacecraft, following up with Galileo, Cassini, and Juno, and leading into the future with Juice and Europa Clipper. Finally, the National Academy’s “Origins, Worlds, and Life: A Decadal Strategy for Planetary Science and Astrobiology 2023-2032” places a journey to an Ice Giant as the highest priority flagship mission to the outer Solar System in the coming decade, with Uranus as the preferred target and an orbiter with an entry-probe as the preferred mission. Probes have been considered essential elements of past and future missions to study atmospheric structure and composition, temperature profiles, and wind speeds. The goal of this paper is to show how measurements from an Earth-based observers’ team can substantially enhance the results from an entry-probe released into a giant planet. These measurements can also serve as input to site selection and engineering design.

Although planetary entry-probes possess the obvious advantages of capturing measurements and observations at very high spatial resolution, their two prime drawbacks are that they provide a mere snapshot of conditions on a planetary body, and that they obtain measurements over a small region of a giant planet. To expand their temporal and spatial reach, and to augment knowledge of the physical conditions of their in situ measurements, ground-based observations are key. These observations also provide additional spectral coverage and viewing geometry. Of the outer planets, only Jupiter has been explored by an in situ probe (Young et al. 1996) with an extensive ground-based campaign (Orton et al. 1996, 2023), but the Huygens probe at Titan was surrounded by a similar extensive ground-based campaign that assessed the conditions of the entry point and the encompassing region and that could serve as a model for a planetary probe to Saturn, Uranus, or Neptune (Witasse et al. 2006). Other events, such as the Shoemaker-Levy 9 cometary impact into Jupiter and the Lunar Crater Observation and Sensing Satellite (LCROSS) impact onto the Moon are “probe-like” and profited from robust ground-based campaigns (e.g. Orton et al. 1995).

Table 1 illustrates the complementary nature of Earth-based and mission/in situ measurements. Quantitative comparisons for viewing geometry and spatial resolution depend on the target, the mission, and the ground-based asset; the Table provides constraints that are defined by fixed geometric configurations and existing capabilities so far. The solar phase angle (the angle between the Sun, Earth, and observer) is limited to very small angles for the outer Solar System: the outer planets are observed fully or nearly fully illuminated from Earth so that the forward-scattering of hazes and the atmosphere can never be observed. Missions such as Voyager, Galileo, Cassini, and Juno provided observations at larger solar phase angles and higher spatial resolution (Smith et al. 1986, 1989; Banfield et al. 1998; Porco et al. 2005; Flasar et al. 2005; Bolton et al. 2017) while Earth-based assets provide long-term temporal evolution, additional wavelengths, and comprehensive modeling (Simon et al. 2015, 2021; Irwin et al. 2012, 2024; Orton et al. 2014, 2015, 2023; Chavez et al. 2023; Sromovsky et al. 2019, 2001). Although small phase angles can be observed by missions, design constraints generally preclude detailed measurements of them except for long duration missions (e.g., Buratti et al. 2022); these observations are best obtained by Earth-based assets for the outer Solar System as they represent the usual configuration of the Sun, target, and Earth (e.g., Buratti et al. 2021).

**Table 1:** Viewing geometry and spatial resolutions available for the outer planets from Earth and space

Target	Phase angle range from Earth^*^	Earth-based spatial resolution^**^	Best space-based resolution	Comments and Citations
Jupiter	<12°	70 km	1 km; Juno, Jovian Infrared Auroral Mapper Camera (JIRAM)	Adriani et al. (2017)
Saturn	<6°	145 km	∼0.5 km, end of Cassini mission	https://science.nasa.gov/missions/cassini-/cassini-top-images-2017/
Uranus	<3°	310 km	Voyager 2; ∼20 km	Best resolution on rings; planet resolution was ∼200 km; Smith et al. (1986)
Neptune	<2°	513 km	Voyager 2; ∼20 km	Smith et al. (1989)

^*^Full range of solar phase angles is possible from a spacecraft, depending on mission design

^**^0.1”/pixel optical resolution of JWST

Entry-probe measurements provide “ground truth” for remote observations. Having in hand the combination of simultaneous in situ and remote measurements means future remote observations can be more robustly compared with the physical conditions that exist on the planet. The importance of a ground-based observing program to accompany a transient planetary mission – a flyby or a probe entry – was realized at the time of the 1973 flyby of Jupiter by Pioneer 10, the first space-based mission to explore this planet (Coffeen 1973).

The assets, teams, and strategies developed for a wide range of ground-based observing campaigns are similar to those expected for a giant planet entry-probe, which is the subject of this paper. Thus, we summarize the types of observations that have driven a selected set of these teams to provide a general resource for those forming future teams, with a focus on those designed for giant planet probes. International partners and amateur collaborations can expand the reach of a ground-based observing campaign. International collaborations give access to a greater array of facilities, expanding the range of observing times and geometries. Amateur astronomers and citizen scientists can also obtain valuable observations, especially if a member of the flight team organizes efforts to observe in the right spectral bands and at the right times. Alerting amateur teams to unexpected events and immediate follow-ups often provides key temporal coverage, as in the example of jets occurring on 67/P Churyumov-Gerasimenko (Snodgrass et al. 2017). In addition, amateur astronomers, being an essentially around-the-clock resource, can often alert mission scientists to transient events that can be observed by spacecraft teams at much greater spatial resolution.

Also key are comparisons with past missions. For example, Beebe et al. (1996) compared Galileo probe measurements of Doppler wind speeds with earlier results derived from the Hubble Space Telescope (HST) and Voyager, as described below. Data from these past missions, as well as telescopic data, can be used as calibration sources for probe data and spacecraft measurements in general, due to the well-calibrated sources that astronomers use.

Finally, ground-based observations are uniquely different from spacecraft operations, as summarized by Witasse et al. (2006; Table 3). They are low-cost and involve a large segment of the community, such as amateur astronomers and citizen scientists who are well-connected with the traditional conduits of public outreach and education such as astronomy clubs, NASA Solar System Ambassadors, and K-12 schools. In the case of a mission failure, ground-based observations can provide backup information – such as tracking data – to assess the circumstances of the failure. Ground-based programs also include experiments that can only be accomplished from the Earth, such as very-long baseline interferometry (VLBI), a technique that can be used to track the coverage of a planetary probe (Folkner et al. 1997, 1998; Witasse et al. 2006). Analysis of the signal accurately tracks the position of the probe to assess the circumstances of a potential failure, but this same signal can also collect scientific data such as horizontal zonal wind speeds. Finally, there are unique viewing geometries that are only observable from the Earth.

Ground-based campaigns to support probe science come in two flavors: those that are organized to play out over many years, and those that are focused on the entry event. The former enlarge the temporal excursion to place observations from a probe within the context of transient events, longer-term seasonal changes, and the big spatial picture, while the latter are designed to understand the conditions centered on the probe entry site and its location. Past experience suggests that organizational efforts to consolidate teams dedicated to support of probe-entry science should form at least 18-24 months prior to the event and that observers should plan to obtain data not just at the entry site, but in adjacent regions, especially in the downstream wake of the probe (Lorenz et al. 2007).

Astronomical observations of an outer planet atmosphere go far beyond the data that is gathered: data collected through space and time yield a physical model of the atmosphere that provide planning guidelines for probe entry and context for the results. Recently NASA has underscored the development of atmospheric models for missions to the outer Solar System that utilize aerobreaking capture into orbit (NASA 2023). Much of the rationale for the development and application of these models is applicable to the probe-entry phase of a mission as well. Often ground-based and spacecraft data provide key regions of a spotty data set that, when combined with physical modeling, can infer physical conditions such as molecular or elemental abundances or temperature and pressure conditions in areas that were not measured. In other cases, archived data can be accessed and analyzed to provide decades-long tracking of spacecraft targets. For example, recent analyses of archival Neptune data have revealed significant variability in atmospheric conditions, including unexpected and dramatic drops in stratospheric temperatures and cloud coverage in recent years (Roman et al. 2022; Chavez et al. 2023). Such studies place planetary probe results into the context of long-term weather patterns, including seasonal changes, and cloud motions.

Archived data sets comprise two types: those more recent ones such as those of HST, the James Webb Telescope (JWST), and planetary missions, and those obtained over many years at observatories and with search programs. The former is more useful for probe site-selection, but the latter can be used to seek unique temporal phenomena, such as the occurrence of volcanic activity or a storm system, which would be a good candidate for a probe entry. Working in a support team’s favor is NASA’s recent policy of requiring that all peer-reviewed publications include archiving of the original data.

Observational support programs are key components of planning and executing a probe entry into a giant planet, but it is still important to plan measurements from the associated orbiter if there is one and if it is technically feasible. Such observations are obtained at much higher signals and spatial resolution (especially for the ice giants), and they would provide detailed information in the case of a failure.

All flight missions can benefit from ground-based support campaigns, largely for the same reasons outlined here for missions to the outer planets that include an entry-probe to the primary Many missions do not figure in the cost and administrative effort of an observer support team, so most support astronomers rely on their own funding. NASA’s Solar System Observations Program (SSO) explicitly funds ground-based support of missions. In the future, missions should plan on having a person who would be cognizant of the details involved in leading such a team, and allocate funds to partially support the observing program. These funds would be a small part of the budget, with a large payoff. In addition, the establishment of a standing observing team forges a path for projects to capture directors discretionary time for follow-up in the case of a transient event such as a cometary jet or an active plume on Europa.

## Past Campaigns

### Galileo Probe

The Galileo Entry Probe Mission was the first in situ probe to an outer giant planet, providing information on its composition and chemistry (Niemann et al. 1996), zonal wind speeds and turbulence (Atkinson et al. 1996), thermal structure (Seiff et al. 1996), cloud and lightning properties (Ragent et al. 1996), particle and radiative environment (Fischer et al. 1996), and helium abundance (von Zahn and Hunten 1996) during its entry into the Jovian atmosphere on December 7, 1995 (Young et al. 1996). Important to its success were extensive ground-based campaigns, which are summarized in Orton et al. (1996). Among the key discoveries were that the zonal winds extend deep into Jupiter’s atmosphere, that the helium abundance was greater than expected, similar to solar abundance, and that both neon and oxygen were depleted. These observations served to create a bigger picture for the results returned by the probe, and to explain some of the unexpected results.

**Fig. 1:**
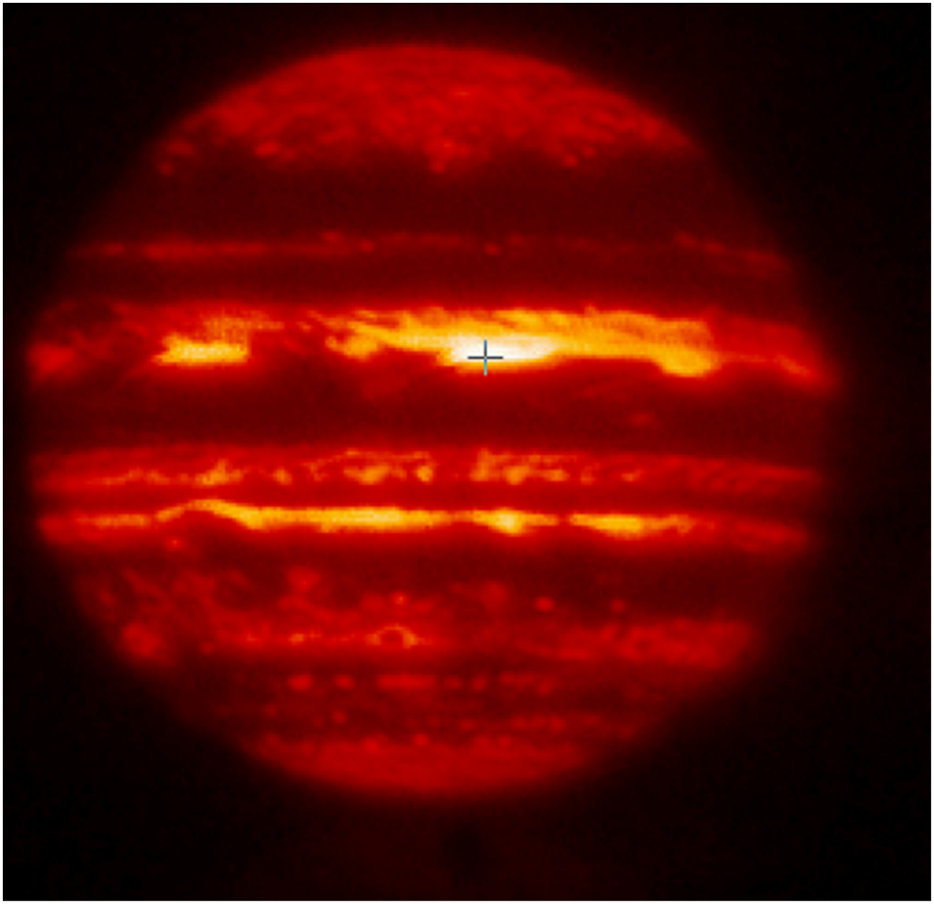
An infrared (4.85 μm) image obtained of Jupiter at the IRTF 16 days before the entry of the Galileo probe. The X is the location of the probe’s entry. Based on Orton et al. (1996)

Orton et al. (1996) describe a large team consisting of observers on HST covering the 0.255-0.953 μm region, at several near-infrared facilities including the NASA Infrared Telescope Facility (IRTF), Kitt Peak, and Sacramento Peak, at the thermal infrared cameras at Kitt Peak and IRTF, and with CCD cameras at Kitt Peak, Pic-du-Midi Observatory, the Donald C. Parker Observatory, the Swedish Solar Observatory, and Yerkes Observatory. In addition to providing spatial context for the probe entry (see Fig. 1), the team was able to determine that the entry site was far from typical. It was drier than 99% of the planet with downwelling gas and a dearth of particles larger than a micron (Niemann et al. 1996; Atreya et al. 1996; Showman and Ingersoll 1998). These findings explained the relatively low abundance of particulates found by the probe (Ragent et al. 1996). Longitudinal wave structures inferred by ground-based observations also provided context for the probe’s vertical temperature profile. Because of Jupiter’s mere 9° distance from the Sun at the time of the probe entry, this suite of observing platforms was dominated by solar telescopes and special filters covering the entire IRTF mirror that allowed such observations to take place safely.

Although the Galileo probe was not designed to be tracked by Earth-based telescopes, the 27 antennas of the Very Large Array (VLA) in Socorro, New Mexico provided an effective collecting area of a 130 M antenna, and the six antennas of the Australia Telescope Compact Array (ATCA) in Narrabri, Australia provided an effective 54-m diameter telescope (Folkner et al. 1997). While the signal detected at Earth was only one billionth of that detected by the Galileo orbiter, the two data sets agreed qualitatively. Furthermore, this experiment laid the groundwork for future probe descents, most notably the Huygens probe entry described below.

Reconciliation of disparate results obtained by spacecraft and ground-based teams can often extend knowledge to times and places not observed by either team, especially when combined with modeling. A good example of this tack is illustrated by ground-based radio observations of the abundance of ammonia in the atmosphere of Jupiter, which shows solar-type abundances at pressures greater than 3 bar, and subsolar abundances at pressures less than one bar (de Pater et al. 2001). Galileo probe measurements show much greater abundances of 3.6 times the solar abundance in deeper levels, where pressures exceed 8 bars (Folkner et al. 1998). Reconciliation of these measurements established the global transition zone where Jupiter’s ammonia abundance falls below solar values to be < 2 bars, with substantial latitudinal differences occurring (de Pater et al. 2001).

In another example, Beebe et al. (1996) compared Doppler wind speeds of Jupiter’s equatorial clouds measured by the Galileo probe with translation speeds derived earlier from HST and Voyager data. Discrepancies in the two data sets led to the idea that the smaller wind speeds measured earlier were characteristic of large weather systems while the larger wind speeds (∼103 m/sec) measured at the probe site were related to localized jet streams. These jets were always there, but they were masked by the larger systems.

### Huygens Probe

The Huygens probe was a Titan atmospheric entry probe attached to the Cassini mission with a payload consisting of the Huygens Atmosphere Instrument (HASI), the Doppler Wind Experiment (DWE), the Descent Imager/Spectral Radiometer (DISR), a Gas Chromatograph Mass Spectrometer (GC/MS), an Aerosol Collector and Pyrolyser (ACP), and a Surface Science Package (SSP) (Lebreton and Matson 2002). The probe was jettisoned from the main Cassini spacecraft on December 25, 2004, landed successfully on the surface of Titan on January 24, 2005, 2 hours 30 minutes after atmospheric entry. The probe continued to transmit data on the surface properties of Titan for 54 minutes, relayed to Earth via Cassini’s radio antenna. Although not a giant-planet entry-probe, Huygens scientific goals were centered primarily on the atmosphere of Titan, and its payload would be similar to a probe focused on the study of a giant planet’s atmosphere (and of course it was part of a mission to a giant gas planet). Finally, the Huygens probe was accompanied by a large, all-encompassing team of seasoned observers, providing a model for future teams (Witasse et al. 2006).

A key component of the Huygens observing campaign was its radio-tracking effort: 17 Earth-based telescopes were involved in a plan to detect the signal from the probe at 2040 MHz; to augment the results from DWE by creating a complete two-dimensional view of the horizontal wind field along the probe’s path, and to determine the landing position of Huygens. This effort relied on the heritage of the successful Galileo campaign (Folkner et al. 1997) described above, and on previous campaigns with substantial improvements in the capabilities of very long baseline interferometry (VLBI) resulting in milliarcsecond accuracy in the emitting position (Witasse et al. 2006).

Observations in the visible and infrared regions of the spectrum were obtained at 11 Earth-based or Earth-orbiting telescopes in support of the Huygens Probe entry. Six near-IR and optical (0.8-1.0 μm and 1.2-2.5 μm) telescopes focused on atmosphere and surface characterization (windows in Titan’s atmosphere in this spectral range offer some clarity to the surface), while two telescopes with spectral ranges of 0.42-0.62 μm and 12 μm measured the zonal wind and ethane profile. The campaign lasted from December 18, 2004 until February 28, 2005. Corroborating evidence and global context provided during this time included evidence for a temperature inversion layer in the stratosphere; three-dimensional measurements of zonal wind speeds and haze; atmospheric density and composition; surface albedo; and cloud activity in the time leading up to the probe descent (see review by Witasse et al. 2006). Attempts to detect thermal or spectral signatures of the Huygens probe entry were made at five telescopes, but nothing was detected (Lorenz et al. 2007). However, this study highlighted several improvements for future similar attempts, included searching for phenomena away from the entry point, such as chemical and thermal changes along the entry-path.

In addition to real-time support, there were longer term observations beginning in 2003 that included a stellar occultation campaign to characterize the atmosphere and cloud activity. There were, of course, many other observations of Titan that were not a formal part of the observing campaign but that provided longer temporal context for the results from the Probe (e.g., Schaller et al. 2005)

### Probe-Like Event: Shoemaker-Levy 9

The impact of S-L 9 into Jupiter in 1994 was a far more energetic event than a probe release, and of course the impactor lacked in situ instruments: the only data were those measured on the ground, in Earth-orbit, or by existing spacecraft. However, the extensive suite of ground-based observers focused on the event provides a model for such a team observing the entry and descent of a planetary probe, as well as clues to possible expected phenomena. Extensive observations from the IRTF, the Wide Field Planetary Camera 2 (WFPC2) on HST, and the W. M. Keck Telescope tracked the thermal response of Jupiter to the event (Orton et al. 1995), the break-up of the fragments of the comets, release of dust and aerosol particles, and emission of magnesium, silicon, and iron ions, sulfur-bearing compounds, and ammonia (Feldman et al. 1996; West et al. 1995; Noll et al. 1995; Graham et al. 1995; Weaver et al. 1995), and the response of the atmosphere and Jupiter’s aurora to the impact (Hammel et al. 1995; Clarke et al. 1995; Prangé et al. 1995). Spectra and lightcurves were obtained at Palomar Observatory (Nicholson et al. 1995a, 1995b). The response of Io’s torus to the impact, particularly the evolution of sulfur and oxygen ion emissions, was observed at the Las Campanas Observatory, by the Ulysses spacecraft, and by the International Ultraviolet Explorer (McGrath et al. 1995).

In addition to ground-based and Earth-orbiting telescopes, the Galileo spacecraft enroute to Jupiter at a distance of 240 kilometers trained its camera, Near-IR Imaging Spectrometer (NIMS), Photopolarimeter Radiometer (PPR) experiment, and Ultraviolet Spectrometer (UVS) on the planet to observe the impact of six fragments – G, H, K, L, Q1, and W (Carlson et al. 1995; Martin et al. 1995; Hord et al. 1995). These events were not observable from the Earth.

### Other Observing Campaigns

Other “probe-like” missions that have involved observing teams include the Lunar CRater Observation and Sensing Satellite (LCROSS^1^); the Double Asteroid Redirection Test (DART; Thomas et al. 2023), and the joint ESA-NASA mission Rosetta, which tracked the temporal behavior, including that of jets, of the comet 67/P Churyumov-Gerasimenko through a perihelion passage and included the efforts of amateur astronomers (Snodgrass et al. 2017).

### Current Observing Campaigns

From its outset, the Juno mission fostered and supported the coordination of a continuous set of measurements of the Jovian system in order to expand and enhance the value of the data collected from its own suite of instruments. These Earth-based measurements, including those from ground-based and spacecraft platforms, cover a suite of instruments providing broad spectral coverage from the X-ray through the radio. Besides expanding the spectral coverage of Juno’s instruments, they provide measurements of the spatial context of the often very narrow regions of the planet that Juno’s instruments cover, as well as a means to determine the evolutionary history of features. Their coverage includes not only Jupiter’s atmosphere, but also its magnetosphere via characterizations of auroral phenomena that are made contemporaneously – often simultaneously – with Juno’s particle and field in-situ measurements. A summary of the observations made by each orbit is publicly available, which mirrors an interactive site that is populated by the observers and curated by members of the Juno science team.^2^ Taking its lead from the Juno mission, the Europa Clipper mission has also initiated a ground-based observers’ group. Although neither of these missions carries a probe, their observing teams are very similar to that for a probe-mission in terms of equipment and the scientific expertise of the participants.

## Modeling

Atmospheric models that are based on years or decades of conglomerated mission and Earth-based observations as well as archived data are key resources for the development of both engineering requirements and scientific goals for probes entering the outer planets. Global Reference Atmospheric Models (GRAMs) provide foundational information on physical characteristics such as composition, wind speeds and direction, and variability that provide the basis for selection of a probe site and an understanding of its environment. NASA’s Science Mission Directorate recently commissioned an Aerocapture Demonstration Relevance Assessment Team (ADRAT) to “examine the utility of an aerocapture demonstration mission to reduce the risk on outer planets missions, focusing on how NASA can use this technology to more effectively meet the nation’s science and exploration goals” (NASA 2023). Although this study was concerned with questions and requirements surrounding aerobraking technology, many of its recommendations are applicable to the preparation for a probe mission to the outer planets. In addition, the main spacecraft that delivers the probe may be captured by aerobraking technology: ground-based observations and modeling will serve a dual purpose for such missions. One key conclusion of this report is that the main risk to aerocapture of a spacecraft is “uncertainty in the destination’s atmosphere and environment”. The selection of a probe entry site will have the advantage of reconnaissance by the parent spacecraft, but GRAMs will provide the long-term context.

The ADRAT report emphasized the need to conduct long-term (decades) synoptic observations on the giant planets (Jupiter, Saturn, Uranus, and Neptune) and Titan, and simultaneously to make use of archived data. These aggregated data sets were used to uncover, for example, unexpected periodicities in Jupiter’s atmosphere that were not coupled with seasonal changes (Orton et al. 2023), stable temperature distributions on Uranus between 1986 and 2011 (Orton et al. 2015), and the modulation of Neptune’s cloud activity with that of the Sun (Chavez et al. 2023). The report recommended continuing the Outer Planet Atmospheres Legacy (OPAL) program to generate yearly global maps of each of the outer planets (Simon et al. 2015, 2021; see below), and it also recommended that a similar program be implemented on JWST. Occultations of cosmic radio sources should also be considered for probing atmospheric levels deeper than those accessed by stellar occultations.

In addition to the peer-reviewed literature that describes models generated from ground-based and spacecraft data such as those for Saturn (Koskinen et al. 2021), Neptune (Ingersoll et al. 1995), and Titan (Coustenis 2005), NASA maintains a set of online GRAMs for Uranus, Neptune, and Jupiter (Justh et al. 2020, 2021a, 2021b)

## Summary of Assets for a Probe Campaign

Astronomers organizing teams of observers to track entry probes and probe-like events have tended to use the same assets and make the same types of observations, which are summarized in Table 2. Our intent is for this summary to serve as a checklist for organizing ground-based support campaigns for probe entry events and for missions in general.

**Table 2:** Summary of assets by type with scientific goals

Asset	Examples (not exhaustive)	Scientific contributions
Large and medium-sized optical telescopes	W. M. Keck/Very Large Telescope (VLT), Gemini, Subaru, Palomar (AO) Telescopes.	Context; temporal extension; additional viewing geometries; more complete wavelength coverage; calibration; planning observations
	Future: Giant Magellan Telescope (GMT), Roman, Thirty Meter Telescope (TMT), European Extremely Large Telescope (ELT)	
Infrared telescopes	IRTF; Palomar (Triple Spec), Gemini, Subaru	Entry markers; atmospheric conditions; composition of region entered
Thermal Infrared telescopes	IRTF, JWST, VLT, Subaru	Thermal signature of entry; ambient temperature
Medium-sized and small telescopes	Table Mountain Observatory; University telescopes; Some amateur telescopes	Same as for large telescopes; greater ease of obtaining time may offset lower spatial resolution; good for temporal coverage and large fields (e.g., Io torus)
Space-based	HST, JWST, International Ultraviolet Explorer (IUE)	Reconnaissance, site-selection, and observational planning; extended spectral coverage; temporal context
Radio telescopes, including VLBI arrays	Greenbank; Very Long Baseline Arrays at various locations; Atacama Large Millimeter/submillimeter Array (ALMA)	Tracking of probe through atmosphere; atmospheric conditions; failure analysis; images at a unique wavelength
Radar	Goldstone; Greenbank; Haystack	Size determination of target; more appropriate for Near-Earth Object (NEO) missions such as DART impact
Exotic assets	Chandra X-ray and XMM-Newton telescopes	Unexpected discoveries such as the discovery of X-rays at Pluto (Lisse et al. 2017)
Existing spacecraft, including parent vehicle	Galileo observation of S-L impact Juice&Europa Clipper collaboration	Synergistic science, different geometries, effective data relay
Amateur telescopes	Rosetta and Juno missions	Context, follow-up of transient events; occultations; outreach and publicity; citizen science

Monitoring programs before, during, and after a probe mission are a critical component of any ground-based observing support program. Many astronomical observers have shown great foresight by designing programs that transcend any plans for a specific mission. An existing data set that extends over years and possibly decades enables the placement of probe results representing only minutes into the context of a much large temporal timeframe. For example, the Outer Planet Atmospheres Legacy (OPAL) program is generating yearly global maps of each of the outer planets (Simon et al. 2015, 2021). Results from this program have extended the temporal behavior of Jupiter’s zonal wind profile and the evolution of its red spot from Voyager and Cassini time periods. OPAL was also able to extend the period of observation of the north polar storm of Saturn that developed in 2018, as well as of other features and wind patterns that developed in its atmosphere. Publicly archived OPAL observations of Uranus and Neptune have also helped to reveal significant long term trends in cloud cover (Karkoschka 2011; Chavez et al. 2023), transient vortices (Hsu et al. 2019a), and the evolution of dark spots on Neptune (Hsu et al. 2019b). It is hoped JWST observations will similarly be used to track temporal variability on the outer planets, ultimately providing valuable long-term context for future missions.

**Fig. 2:**
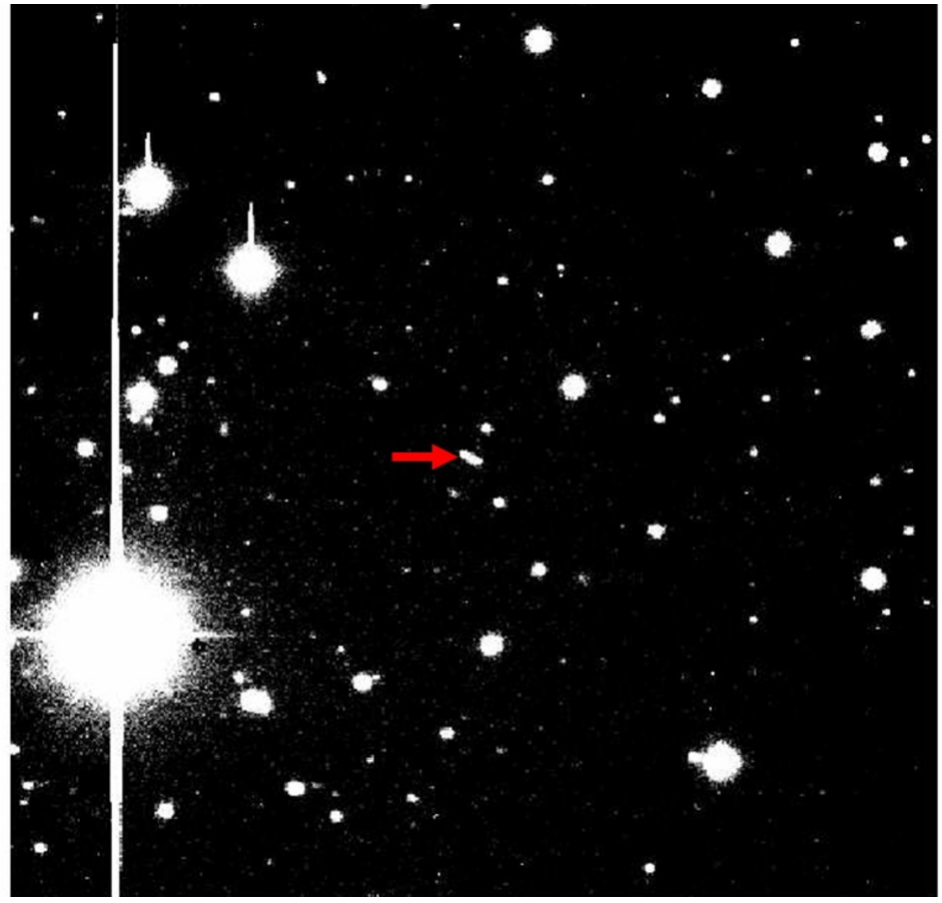
An image from the Hale Telescope at Palomar Mountain Observatory of the Deep Space 1 spacecraft obtained on November 16, 1998, 23 days after its launch from Cape Canaveral. The spacecraft is marked with a red arrow. Observers were B. Buratti, A. Doressoundiram, M. Hicks, and P. Weissman

Outreach activities and observing by citizen scientists – especially during key periods such as launch, cruise flybys of asteroids or other targets, and gravity assists during cruise – can lead to focused E&PO events that include lectures, star parties, educators’ workshops, and news releases, as well as positive early publicity for a mission. Images of spacecraft right after launch also provide a vehicle for sparking continuing interest in a mission right after the time of launch, an afterglow to the first event that gives substantial publicity to a mission. Since probe investigations are generally associated with flagship missions, it is possible for amateurs and astronomy clubs to obtain post-launch images, potentially forming the basis of education and outreach events early in interplanetary cruise. “Planet watches” during the probe release also offer high popular interest and value. Of course, nothing can be seen of the probe itself, but accompanying commentary and telemetry from NASA TV would form the basis of a public event attracting hundreds of people. Figure 2 shows an image of the Deep Space 1 spacecraft obtained shortly after launch with the Hale Telescope at Palomar Observatory. With larger (brighter) flagship spacecraft, amateur telescopes could capture similar images, especially if they are obtained soon after launch.

## Observations for Future Campaigns

### The Uranus Orbiter and Probe

The Decadal Report assigned the highest priority flagship mission to a Uranus orbiter and probe (UOP) with a recommended start of phase A in FY 2024. Not only does this ice giant present profound questions concerning its atmosphere, rings, magnetic field, and large axial tilt, it is potentially representative of the largest class of exoplanets of similar size. The mission envisioned by the report would entail a multi-year orbital mission involving a study of the planet, rings, and moons, as well as the delivery of an atmospheric probe into the Uranian atmosphere. Because the annual cycle on Uranus is large – 84 years – a temporal baseline of seasonal changes, including possible volatile transport on their moons, should be decades long. In addition, clarification of the scientific goals of the mission and instrument requirements would benefit from ground-based studies on the presence of specific volatiles and minerals and their distribution. In general, the types of observations would closely follow those implemented for the Galileo probe mission, with the addition of more advanced assets such as JWST, ALMA, VLAs for tracking the probe, and the next generation of larger telescopes that will be coming online in the next decade (e.g. the Extremely Large Telescope (ELT), the Thirty Meter Telescope (TMT), and the Giant Magellan Telescope GMT)). The JWST planetary programs awarded so far cover topics that address change on the giant planets and monitoring, including transient events and temporal variability on Neptune.^3^

Images from JWST^4^ show details of cloud dynamics on Uranus and Neptune plus an intriguing change in the color of Uranus, but these data are currently just momentary snapshots of the long and unresolved seasonality of the outermost planets that will be substantially extended as future observations are made and archived. Supplementing these high resolution images with images from large Earth-based telescopes, especially those with adaptive optics systems, would enable a view of temporal changes at a much more granular scale. In addition to providing reconnaissance for probe-entry site selection and long-term context for the site, detection of changes in the optical depth and clumpiness of the Uranian ring system would be especially valuable for planning observations for the mission, as well as for yielding a clear picture of the dynamics of the ring-system and any interactions of it with possible ring moons existing within the ring or in its vicinity. Images that include satellites could map out the distribution of volatiles on their surfaces, results that could in turn be used for mission planning. Although single images of these companion moons would contain little information on this distribution, images at different latitudes or longitudes could do so, in a fashion similar to the way rotational light curves reveal different volatile abundances through absorption-band strengths at various sub-observer locations. Such techniques have been applied to the moons of Uranus (Grundy et al. 2006), and tracking of the seasonal transport of volatiles on Triton (Hicks et al. 2022) has provided a long temporal handle for modeling of seasons in the outer Solar System.

**Fig. 3:**
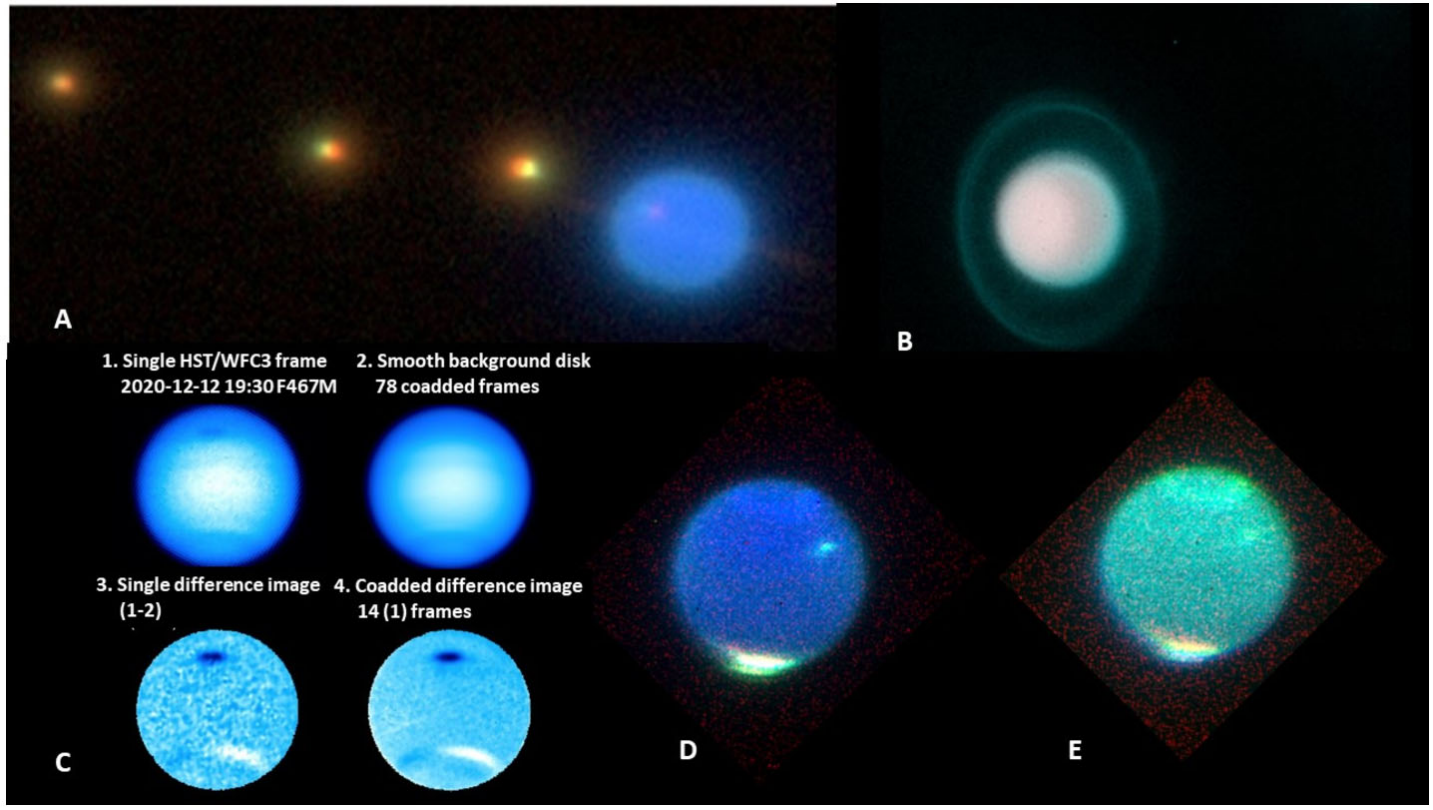
Various images of the ice giants, including some obtained by the authors. **A.** An image of Uranus obtained with the Palomar Adaptive Optics system on the Hale Telescope on 20 September 2007, with 1.55 μm, 1.65 μm, & K-band in the RGB bands. Three moons and the rings are visible. **B.** An image of Uranus obtained with the same equipment on July 19, 2021, with the J-filter image in the R-Band, the CH_4_ long band image in the G-band, and the CH4 short filter image in the B-band (the CH_4_ long band filter has a spectral range of 1.64-1.74 μm and a central wavelength of 1.69 μm, while the CH_4_ short band filter has a spectral range of 1.52-1.62 μm and a central wavelength of 1.57 μm). **C.** Visible (0.47 μm) images of Neptune obtained by HST in 2020 tracking the evolution of a dark vortex (Space Telescope Science Institute 2022). The headings describe a co-adding and difference scheme to accentuate the feature. **D**–**E.** Images of Neptune obtained with the Palomar Adaptive Optics system in July 2021. The left image has the JHK filter images placed in the RGB planes, while the right image has the CH_4_(short), CH_4_(long) and K(short, with a central wavelength of 2.145 μm and a range of 1.99-2.30 μm) placed in these planes. These images suggest the possibility of doing compositional analyses (as described in the text) as well as tracking of cloud formation and motion through a seasonal cycle

Any support program that includes a long temporal baseline will contain a few images at high spatial resolution, some at medium resolution, and many at low resolution. Moderately-sized telescopes can contribute significantly to detecting long-term variation in cloud cover while practically ensuring greater access as a result of lower subscription/demand (e.g., Roman et al. 2018). Figure 3 provides a few of the types of images expected at medium resolution for an ice giants mission. Many of these images were obtained in filters that are sensitive to the abundance of volatiles such as methane, water, and CO_2_. Mapping the depth of absorption lines for these materials would indicate relative abundances on both the primary and its moons. Images and spectra obtained over years and decades would provide the temporal context and changes for a probe mission.

## Summary

An Earth-based observers’ support program coordinated with an entry-probe mission to a giant planet substantially increases the scientific return of the probe event and the mission in general. Ground and orbital-based observers provide spatial and temporal context, additional viewing geometries, enhanced wavelength coverage, tracking data that can be used for scientific analysis and information in case of a failure, and additional opportunities for education and outreach. These advantages are enhanced with the addition of international observers and amateur astronomers. The existence of a standing observers’ team serves the entire mission by providing these same enhancements to observations from the main spacecraft delivering the probe, whether it is an orbiter or flyby-vehicle. Follow-up or precursor studies to transient phenomena are also enabled. Observing teams should be convened under the guidance of the mission project scientist, who may want to allocate modest levels of funding. If funding is not available, observing teams would fund their programs with NASA or NSF funding or resources from their own institutions. Another key line of investigation is to analyze archived data, including observations from past planetary missions. This task has become increasingly easier, as many observatories, HST, JWST, observers’ peer-reviewed papers, and the Planetary Data System (PDS) all provide archival data, much of it calibrated. Although this paper describes the assets and observations for a team assembled for a probe-entry event, similar teams could – and should – be assembled for all interplanetary missions and serendipitous events such as cometary impacts.

## Footnotes

1 https://astrobiology.nasa.gov/missions/lcross/.

2 https://www.missionjuno.swri.edu/planned-observations.

3 https://www.stsci.edu/jwst/science-execution/approved-programs/general-observers.

4 https://www.nasa.gov/solar-system/nasas-webbscores-another-ringed-world-with-new-image-of-uranus/, https://www.nasa.gov/solar-system/newwebb-image-captures-clearest-view-of-neptunes-rings-in-decades/.
